# The Inebriates Bill

**Published:** 1898-06-04

**Authors:** 


### MODERN SOCIOLOGY.

### THE INEBRIATES BILL.

THE agitation against the existing prison system, culminating in the Bill for General Prison Reform that has just been laid before a Committee of the House, has brought out in strong relief the ignorance of the public respecting the practical working of that same system as it is now carried out. Many of the new rules, over which the agitators are very jubilant at present, considering them to be the results of the cry for reform, have, in fact, been in active operation for some time past, and will cause no necessity for any change whatever. Mr. E. Ruggles-Brise, who was invited by the American delegates at the Prison Congress of Paris, in 1895, to visit the United States and study their penal system, has lately submitted a most valuable report on the subject to the Home Secretary; in it he makes use of the very telling expression that there is, in some respects in America, a tendency to flirt with crime. There can be no doubt that a strong inclination to this species of flirtation has been developed in public opinion in our country by the recent agitation, and it seems not unlikely that the same feeling which leads to this inclination to deal lightly with actual crime will also tend to weaken the stern measures which should emanate from the Inebriates Bill if it is to produce any really effective result. Many, indeed, do not stop short of desiring to see an enactment definitely received as law, for the imprisonment of mere inebriates under certain conditions. Doubtless our gentle agitators will consider this a hard and even cruel remedy for the propensity to drink, which is not even classed as a crime; but the fact remains that inebriates are as great a curse to themselves as they are to their country, and it cannot be questioned that any means, however stringent, which can be used for their reformation would, in fact, be a benefit conferred on them, for which they would have much reason to be grateful. The object of the Inebriates Bill, however, falls far short of this, it being merely the enactment of a law for the more effectual treatment of criminal inebriates; yet it involves a problem bristling with difficulties. It is one over which many persons have pondered anxiously, and for the solution of which a variety of expedients have been suggested or attempted. The Scottish Prison Commissioners, for instance, inaugurated a system which decreed "that persons who had been three times imprisoned in one year" for drunkenness resulting in lawless conduct "should on a fourth conviction in the same year be recorded on a local list as habitual offenders, and should then remain on such list for at least thirty months. If during that period they are again convicted, they should, in addition to the usual brief sentence of imprisonment, be committed to an adult reformatory or labour settlement for a term of not less than twelve or more than thirty months."

This plan is said to have proved a complete failure because it did not allow sufficient time for the radical cure of drunken habits. In Ireland a convict farm was established at Lusk, where inebriates and others who had been frequently convicted were intended to work for their own living under some supervision, but it had to be abandoned because it was found that the inmates corrupted each other hopelessly. In America similar experiments have been made, and we believe still are made; but it is an ominous fact that in spite of the princely reformatory at Elmira, with all its elaborate indulgences, crime has of late increased in the United States with alarming rapidity. A system of prison asylums for life as receptacles for habitual offenders of all kinds, on the ground that they may be treated as practically insane has also been proposed, but we feel certain that any such attempt to set aside absolutely the "liberty of the subject" would have most dangerous results. We have seen enough of imprisoned inebriates to have formed some definite theories as to what might be tried for their reform, and it must be understood that it is not for the simple fact of being drunk that any man or woman is sent to gaol, but for the results of their intoxication in disorderly conduct, breaking windows, fighting, assaulting the police, and so on. One main factor in the existence of habitual drunkenness is the inveterate drinking habits which are the invariable accompaniment of the tramp life. It does not seem likely that any legislative measures will ever conquer the indomitable love of a wandering life which possesses the vast army of tramps, and therefore the State will do well to adopt as speedily as may be some mode of dealing with the determined inebriates they supply to our gaols. A period of detention enforced by law for a sufficient length of time to eradicate the craving for stimulants, is undoubtedly the only plan that promises any hope of success; but the difficulty is to find a suitable abode for temporary incarceration. It has been suggested, and with some show of reason, that a domicile admirably adapted for the purpose could be found by simply making use of our union workhouses. The ratepayers in this land keep up these institutions at an enormous expense, only, it is said, to find the poor obstinately refusing to avail themselves of the board and lodging provided for them, from a rooted prejudice which has no ground in reason. The sole drawback they can justly complain of there is the loss of liberty, and that is precisely the advantage eminently to be desired for the habitual drunkard. It is urged that if the State would exercise its judicial powers in enacting that habitual drunkards should be sentenced to confinement in the workhouse for a sufficient term of years to eradicate their ingrained habits, such a system would have the additional advantage of proving to the lowest of our populations that the workhouse is not a parochial hell, but a kindly and comfortable home for infirmity, destitution, and for all who have lost the power of self-control. It is fair, however, to doubt whether such a scheme as this could ever become a practical reality. However great may be the difficulty of passing laws for the regulation of inebriates, we may be quite sure that those difficulties would be increased tenfold by any attempt to combine with them such a complete reorganisation of the Poor Law system as would be involved in a scheme of this kind. For inebriates are by no means necessarily paupers, and the workhouses certainly are not prisons.

